# Radiation‐mediated supply of genetic variation outweighs the effects of selection and drift in Chernobyl *Daphnia* populations

**DOI:** 10.1111/jeb.13983

**Published:** 2022-01-29

**Authors:** Jessica Goodman, June Brand, Gennady Laptev, Stuart K. J. R. Auld

**Affiliations:** ^1^ Biological & Environmental Sciences University of Stirling Stirling UK; ^2^ Ukrainian HydroMeteorological Intstitute Kiev Ukraine

**Keywords:** evolution, ionizing radiation, microsatellites, population structure

## Abstract

Populations experiencing varying levels of ionizing radiation provide an excellent opportunity to study the fundamental drivers of evolution. Radiation can cause mutations and thus supply genetic variation; it can also selectively remove individuals that are unable to cope with the physiological stresses associated with radiation exposure, or non‐selectively cull swathes of the population, reducing genetic variation. Since the nuclear power plant explosion in 1986, the Chernobyl area has experienced a spatially heterogeneous exposure to varying levels of ionizing radiation. We sampled *Daphnia pulex* (a freshwater crustacean) from lakes across the Chernobyl area, genotyped them at ten microsatellite loci and also calculated the current radiation dose rates. We then investigated whether the pattern of genetic diversity was positively associated with radiation dose rates, consistent with radiation‐mediated supply of *de novo* mutations, or negatively associated with radiation dose rates, as would be expected with strong radiation‐mediated selection. We found that measures of genetic diversity, including expected heterozygosity and mean allelic richness (an unbiased indicator of diversity), were significantly higher in lakes that experienced the highest radiation dose rates. This suggests that mutation outweighs selection as the key evolutionary force in populations exposed to high radiation dose rates. We also found significant but weak population structure, indicative of low genetic drift and clear evidence for isolation‐by‐distance between populations. This further suggests that gene flow between nearby populations is eroding population structure and that mutational input in high radiation lakes could, ultimately, supply genetic variation to lower radiation sites.

## Graphical abstract

We examined population genetic diversity across Chernobyl *Daphnia* populations. Measures of genetic diversity, including expected heterozygosity and mean allelic richness, were higher in lakes that experienced the highest radiation dose rates. This suggests that mutation outweighs selection as the key evolutionary force in populations exposed to high radiation dose rates.

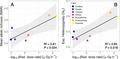

## 1 INTRODUCTION

The explosion of the Chernobyl nuclear power plant in 1986 released approximately 1.85 × 10^18^ Bq of radionuclides into the atmosphere (IAEA, 2006). This radioactive material was deposited over the surrounding landscape in a very heterogeneous manner, leading to radiation dose rates that vary considerably over very small spatial scales (a few hundred metres: Goodman et al., 2019; Shestopalov, 1996). Wildlife populations in the Chernobyl area have thus experienced varying levels of radiation exposure depending on their locations (Hinton et al., 2007). This is potentially important, because high doses of ionizing radiation are known to have strong negative effects on organismal fitness (Barnthouse, 1995; IAEA, 1992; Real et al., 2004), and can cause genetic mutations that can be passed on to future generations in mammals (mice) and crustacean (*Daphnia*) (Adewoye et al., 2015; Parisot et al., 2015). Indeed, a recent meta‐analysis found that increased dose rates of ionizing radiation are associated with elevated mutation rates in Chernobyl bacterial, vertebrate, invertebrate and plant species (Møller & Mousseau, 2015). However, very little is known of how chronic exposure to lower doses of radiation over multiple generations affects the structure and genetic diversity across multiple populations of an individual species (c.f. Baker et al., 2017).

Nuclear accidents such as Chernobyl provide a window through which to view the net outcome of three fundamental processes in evolutionary biology: mutation, selection and genetic drift. Ionizing radiation generates mutations (Adewoye et al., 2015; Parisot et al., 2015) and can thus increase the supply of genetic variation to populations (Haldane, 1937; Kimura & Maruyama, 1966). This is important, because genetic diversity is the currency for both evolution and adaptation (Lande & Shannon, 1996). The dose rates across the Chernobyl area have considerably declined since 1986 because of radioactive decay. Nevertheless, if the current dose rates are sufficient cause mutations, there will be a greater supply of genetic variation at high radiation sites relative to their low radiation counterparts. Long‐term exposure to chronic radiation may also have exerted selection on populations. This could lead to the removal of individuals with inadequate mechanisms for protecting against radiation‐mediated cellular damage (Diehn et al., 2009; Khodarev et al., 2004; Ramana et al., 1998; Smirnov et al., 2012), thus reducing genetic variation more in populations experiencing high radiation dose rates (Schlotterer et al., 1997). Finally, the initial fallout from the accident could have caused bottlenecks across the whole area, indiscriminately depleting diversity and causing non‐selective differentiation, *that is* drift, among populations (Frankham et al., 2002); this would leave its mark in the form of strong population structure (Hartl & Clark, 1997), provided there was low gene flow (Gilpin, 1991; Slatkin, 1987). Indeed, these various evolutionary processes could shape populations in such a way as to reduce fitness differences among them, thus masking the negative effects of ionizing radiation (as we have previously seen in *Daphnia*: Goodman et al., 2019).

Here, we used 10 microsatellite loci to examine the population genetic structure of the freshwater crustacean, *Daphnia pulex*, in seven *Daphnia*‐inhabited lakes in the Chernobyl area. Five of the lakes were within the Chernobyl Exclusion Zone (CEZ), and the other two were situated outside the CEZ. *Daphnia* are perfectly poised to study the effects of radiation because they are known to suffer reduced fitness (Marshall, 1962) and accumulate mutations (Parisot et al., 2015) when exposed to elevated radiation dose rates. Moreover, their populations are defined by the boundary of the water body that they inhabit (i.e. they do not move great distances across landscapes), and the heterogeneous nature of radionuclide deposition across the CEZ provides variation in radiation exposures that are independent of distance between populations (Goodman et al., 2019; Shestopalov, 1996). Migration between populations is therefore limited, though still possible, and gene flow is generally restricted. As we could not explicitly test if radiation dose rate caused shifts in *Daphnia* population genetic diversity, we were able to evaluate whether measures of population genetic diversity across lakes experiencing differing radiation dose rates were consistent with: (1) radiation‐mediated supply of genetic variation, (2) radiation‐mediated selection or (3) genetic drift associated with a massive population bottleneck.

## 2 MATERIALS AND METHODS

### 2.1 Sample collection

Live *Daphnia pulex*, sediment and water samples were collected from seven of the eight *Daphnia*‐inhabited lakes within and immediately outside the CEZ from the 7–16 July 2016. Individual daphnids were immediately stored in 1.5‐ml Eppendorf tubes in absolute ethanol at ambient temperatures and transported back to the UK, where they were stored at −20°C prior genotyping. A total of 205 samples were collected from seven lake populations (27–38 samples per lake; see Table 2). Radiation dose rate was calculated according to the protocol detailed in our previous study (Goodman et al., 2019), where the water and sediment activity concentrations of the different radionuclides were measured for each lake and used to estimate the dose rates to *Daphnia*. In brief, concentrations for the dominant radionuclides in the CEZ (^137^Cs and ^90^Sr) and radionuclides that were considered representative of others present within the water column and top sediment layer (^241^Am and ^239^Pu) (IAEA, 2006) were used to estimate dose rates. Where information was available, data on radionuclide concentrations were extracted from the Ukraine atlas (Intelligence Systems GEO, 2008). Where information was unavailable, water and sediment samples were collected at each sampling site and analysed at the Ukrainian Hydrometeorological Institute (see Goodman et al., 2019).

To estimate dose rates experienced by *Daphnia*, we used the ERICA (version 1.2) software program. ERICA calculates dose rates using an extensive database of published values for radionuclide transfer through the environment and the activity concentrations in various media (lake water, sediment, soil) and organisms, including over 24 000 data entries for freshwater organisms alone (Beresford et al., 2007; Brown et al., 2008, 2016; ICRP, 2009). The user can specify the reference taxon, media and measured radionuclide concentrations, and the tool calculates the average radiation dose rate based on the appropriate subset of data from the database. In this case, Zooplankton was selected as the reference taxon, and the contribution of sediment and water radioactive dose rates was set to 75% and 25% respectively to reflect the period of time *Daphnia* spend in the water column and as dormant eggs in the sediment (see Alekseev & Lampert, 2001). These percentages are conservative as the majority of radionuclides will accumulate in the surface sediment (Nazarov & Gudkov, 2009). It should be noted that radiation is not confounded with lake location; *that is* there is no positive relationship between pairwise distances between populations and pairwise differences in radiation dose rate (Mantel *r* = −0.24, *p* = 0.16).

### 2.2 DNA extraction and microsatellite genotyping

Microsatellite genotyping was used to identify differences in allele frequencies and population structure within and across lake populations following the protocol previously outlined by Auld and Brand (2017). First, genomic DNA was extracted from 205 whole *Daphnia* samples from the seven lake populations (see Table 2 for details) using protocols provided in NucleoSpin Tissue XS (Macherey‐Nagel). We successfully amplified eleven microsatellite markers for each *Daphnia* across two multiplexes (Table S1; Jansen et al., 2011), though one marker exceeded a 5% null allele rate and was thus excluded from further analysis. Multiplex PCRs consisted of 5 µl 2× Type‐it Multiplex PCR Mastermix (Qiagen), 3 µl Nuclease Free H_2_O, 1 µl primer mix solution and 1 µl DNA to give a total volume of 10 µl per reaction. The PCR programme was as follows: 15 min at 95°C for Taq activation, followed by 30 cycles of 30 s at 94°C for denaturation of the DNA into separate strands, 90 s at 57°C for annealing of the DNA strands to template DNA and 90 s at 72°C for extension. The final extension was performed for 30 min at 60°C. The final PCR products were analysed with an ABI 3730XL DNA Analyzer (at the Protein Phosphorylation and Ubiquitylation Unit, University of Dundee, UK) using the GeneScan‐500 LIZ size standard (Applied Biosystems). Microsatellite band scoring was completed manually using GENEIOUS software (Biomatters, version 9.1.8). The strongest peak(s) within the loci were selected to determine allele size.

### 2.3 Analysis

The total number of alleles, mean allelic richness (MAR), the total number of private alleles (PA), and both observed and expected heterozygosities (H_O_ and H_E_, respectively) were calculated (*PopGenReport* package; Adamack & Gruber, 2014, *adegenet* package; Jombart, 2008; Jombart & Ahmed, 2011). Linear models were then used to assess the relationship between log_10_ of the radiation dose rate and each of MAR, H_E_ and H_O_. The index of unbiased association (${\bar{r}}_{D}$; Brown et al., 1980) was then calculated in order to evaluate the level of linkage disequilibrium within populations; this was done using a permutation approach that estimates the levels of recombination in order to detect association between alleles (*poppr* package; Kamvar et al., 2014).

The next step was to test which populations were significantly different from each other. First, an analysis of molecular variance (AMOVA) was used to partition variation within and between populations; the significance of these within‐ and among‐population variation was then estimated using 999 permutations (*ade4* package; Dray & Dufour, 2007; Bougeard & Dray, 2018). We calculated fixation indices (*F*‐statistics; Weir & Cockerham, 1984; Wright, 1951) to quantify the extent of population structure within (*F*_IS_) and among (*F*_ST_) populations (*adegenet* package; Jombart, 2008; Jombart & Ahmed, 2011). Confidence intervals for the *F*_IS_ values for each population were computed by bootstrapping over loci, with 999 permutations using the *hierfstat* package (Goudet & Jombart, 2018). A 999 permutations were used as it eliminates sufficient variation associated with resampling (Hesterberg et al., 2003).

Next, we tested whether populations in close proximity to each other were more similar than those separated by larger geographic distances (i.e. whether there was isolation‐by‐distance). This was done using a Mantel test (implemented using the *ade4* package), which quantified the association between two matrices of pairwise Edward's genetic distances (Edwards, 1971) and pairwise Euclidean geographic distances between populations.

Finally, we conducted a Discriminant Analysis of Principal Components (DAPC) to allow a more comprehensive examination of population genetic structure (implemented using the *adegenet* package; Jombart, 2008; Jombart & Ahmed, 2011). Specifically, we specified *K* = 7 clusters based on our *a priori* knowledge of lake population identity and then used the α‐score procedure to identify the optimal number of principal components (PCs) to retain (in this case, 15). The α‐score procedure maximizes the capacity to discriminate between individuals whilst minimizing model overfitting (Jombart & Ahmed, 2011). The DAPC analysis allowed us to visualize both the clustering of individuals in multivariate genetic space and also evaluate admixture among populations. Following our initial specification of the DAPC model, we tested its performance by dividing the data into two groups: one used for model training (49 individuals) and one used for model testing (156 individuals). We specified an equivalent model with the same number of specified clusters and PCs using the training data, then used the model to assign test individuals to their appropriate populations. By comparing the plot of training and test data, we could evaluate the performance of the model.

## 3 RESULTS

### 3.1 Greater genetic diversity in highest radiation populations

We identified 204 multilocus genotypes (MLGs) among the 205 individuals (Table 2). H_O_ ranged from 0.31 to 0.66 and expected H_E_ ranged from 0.44 to 0.62 (Table 2). Significant linkage disequilibrium was found in Vediltsy, Yampol, Buryakovka and Krasnyansky lake populations (see ${\bar{r}}_{D}$ values in Table 1).

**TABLE 1:** Estimates of genetic diversity among seven *Daphnia pulex* populations at 10 microsatellite loci across Chernobyl

Lake	Estimated lake area	Sampling date	Coord N	Coord E	Upper dose estimate	n	MLG	H_E_	H_O_	A	PA	MAR	*F*_IS_	${\bar{r}}_{D}$
Vediltsy	0.175	07.06.2016	51.4352	30.8385	0.10	27	27	0.46	0.53	30	1	2.66	−0.14	**0.03**
Smolin	0.850	07.06.2016	51.2757	31.0333	0.12	28	28	0.5	0.45	36	2	3.16	0.1	0.02
Yampol	0.020	11.06.2016	51.2095	30.1767	0.20	28	28	0.45	0.39	30	2	2.67	0.14	**0.06**
Glinka	0.005	16.06.2016	51.2174	29.9371	1.17	28	28	0.44	0.31	29	1	2.62	0.3	0.06
Buryakovka	0.350	11.06.2016	51.3978	29.8931	1.77	28	28	0.47	0.33	32	0	2.85	0.3	**0.05**
Krasnyansky	0.066	13.06.2016	51.4429	30.0764	55.79	38	38	0.62	0.5	42	3	3.7	0.2	**0.06**
Gluboke	0.260	13.06.2016	51.4454	30.0653	181.15	28	27	0.6	0.66	43	4	3.68	−0.11	0.02

Note

Estimate lake area is in km^2^ and upper dose estimate is in µGy h^−1^.

A, number of alleles; H_E_, expected heterozygosity; H_O_, observed heterozygosity; MAR, mean allelic richness; MLG, multilocus genotypes; n, number of individuals; PA, number of private alleles; ${\bar{r}}_{D}$, Index of unbiased association (linkeage disequilibrium).

**TABLE 2:** Analysis of molecular variance (AMOVA) assessing the partitioning of genetic variation

Source of variation	*df*	Sum of squares	Variance	% total	*p*
Between populations	6	275.76	0.70	12.52	**0.001**
Within populations	197	1053.84	0.47	8.37	**0.001**
Within samples	204	900.78	4.42	79.11	**0.001**
Total	407	2230.38	5.58	100	

Note

Significant values, resulting from a randomization test of 999 samples, are highlighted in bold.

**FIGURE 1:**
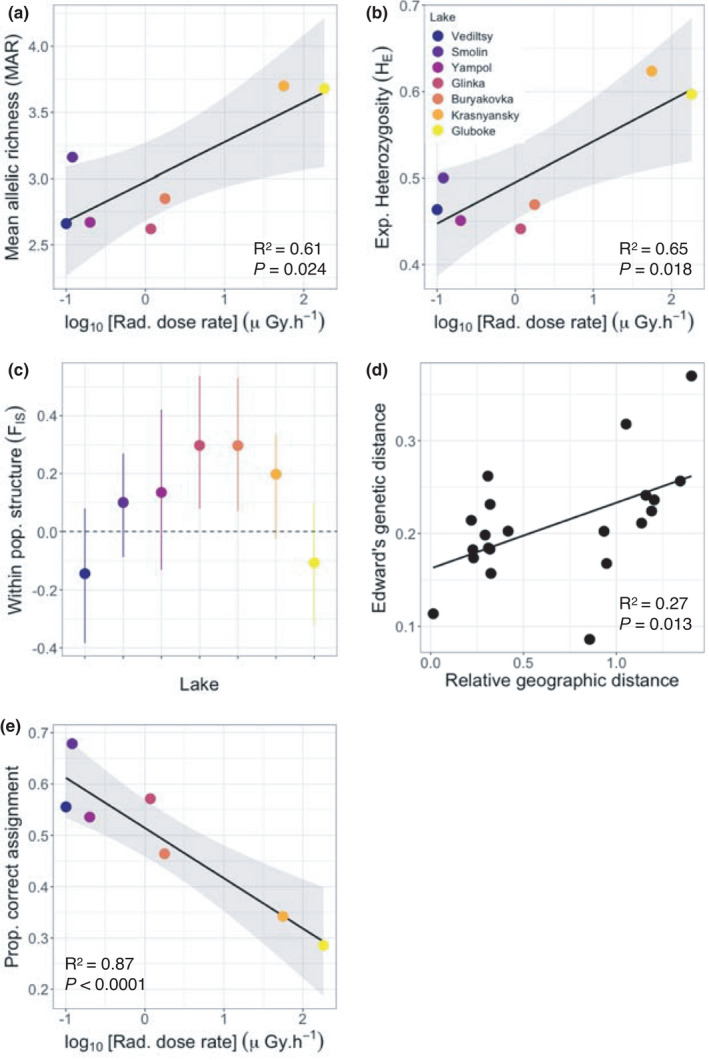
The relationships between (a) mean allelic richness (b) expected heterozygosity and log_10_ dose rate. Points for panels A and B show the raw data, and the shaded area shows 95% confidence intervals from linear models. The *R* ^2^ and *p*‐values are shown for each model fit. (c) Within population structure *F*_IS_ values for each population; 95% confidence intervals were computed using Monte Carlo simulations with 999 permutations. (d) Isolation‐by‐distance plot based upon a Monte Carlo simulation using 999 permutations to test between two matrices of pairwise Edward's genetic distances and Euclidean geographic distances. (e) Likelihood of correct assignment of individuals to lake population based on DAPC analysis

There was a significant effect of radiation dose rate on both MAR (*F*_1,5_ = 10.24, *p* = 0.024; Figure 1a) and H_E_ (*F*_1,5_ = 12.03, *p* = 0.018.); these patterns were clearly driven by the two populations experiencing the highest radiation dose rates (Gluboke and Krasnyansky). We tested the robustness of the relationship between dose rate and MAR by calculating the correlation coefficient (*r* = 0.82) and then performing a randomization test where we compared this correlation coefficient with 5000 resampled correlations where each of the MAR values was assigned a random dose rate value; we found the relationship between dose rate ad MAR to be robust (*p* = 0.024; Figure S1a). We repeated the randomization test for the relationship between radiation dose rate and H_E_, and found this correlation (*r* = 0.84) to also be robust (*p* = 0.037; Figure S1b). There was no effect of radiation dose rate on H_O_ (*F*_1,5_ = 1.67, *p* = 0.25; Table 1). There was no relationship between estimated lake area and either MAR (*F*_1,5_ = 0.18, *p* = 0.69) or H_E_ (*F*_1,5_ = 0.008, *p* = 0.93).

### 3.2 Population structure and gene flow

An AMOVA revealed significant variation within samples, within populations and between populations (Table 2), confirming that there was significant population structure. The overall structure within populations (overall *F*_IS_) was 0.11, and the individual population *F*_IS_ values ranged from −0.14 to 0.30 (Table 1). The lower 95% confidence intervals failed to encompass zero, as is indicative of significant within‐population structure, in two of the seven populations: Glinka and Buryakovka (Figure 1c). The structure across populations was low‐moderate: *F*_ST_ was 0.14, in broad agreement with the 12.52% of molecular variance explained by population (Table 2). However, all pairwise *F*_ST_ comparisons were significant (*p* = 0.001 in all cases), demonstrating that even though there was evidence of gene flow (see later results), each population was indeed a separate entity.

**FIGURE 2:**
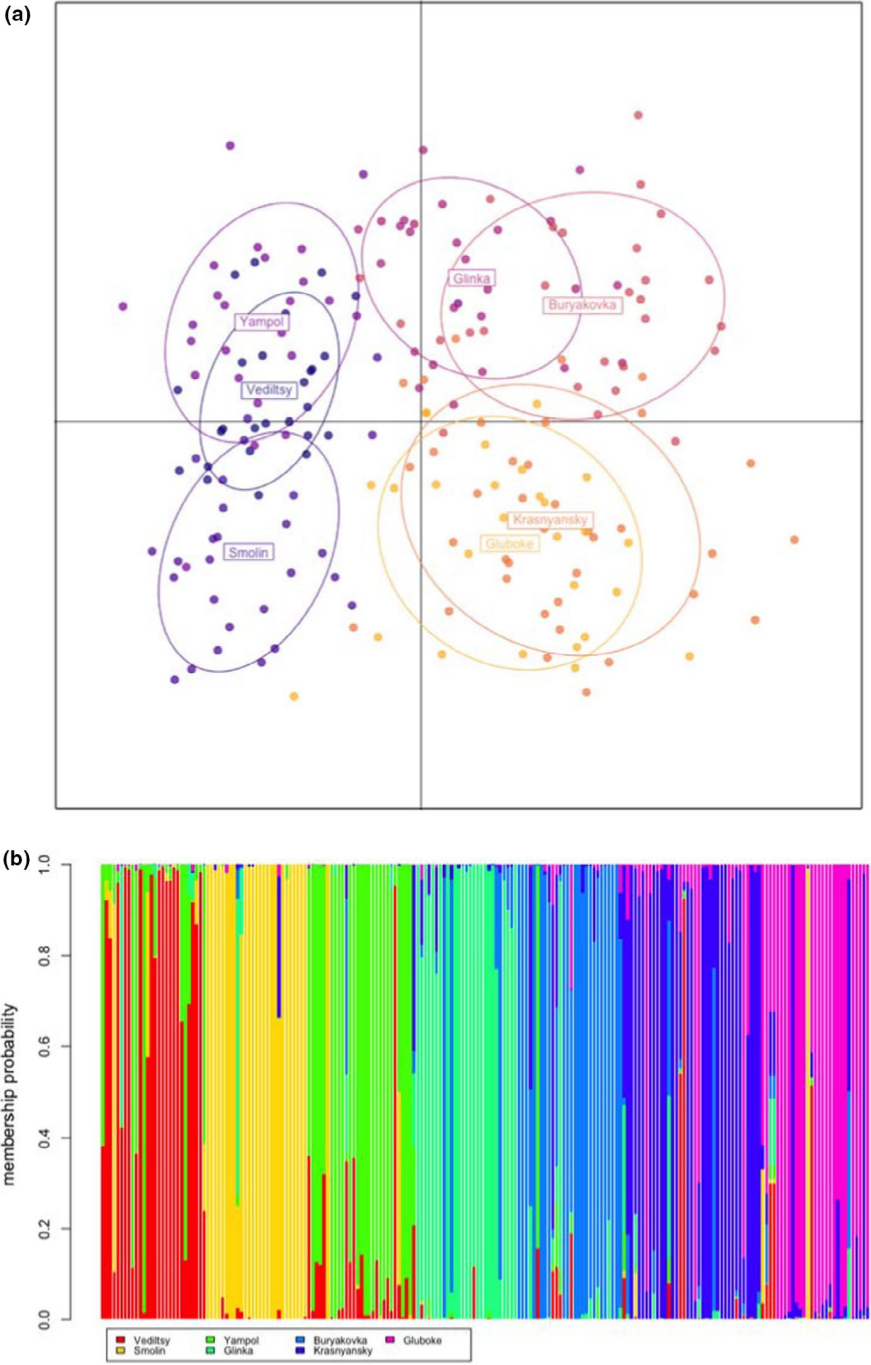
DAPC of the multilocus microsatellite genotypes of 205 *Daphnia magna* from Chernobyl. (a) Plot of individuals in first two discriminant functions, where colours denote population ID. (b) Structure plot demonstrating population assignment

We also found a significant relationship between genetic and geographical distances, *that is* an isolation‐by‐distance effect (Mantel *r* = 0.52, *p* = 0.013; Figure 1d; see Table S2 for pairwise distances between lakes). When we clustered individuals using DAPC, it was clear that whereas individuals generally clustered according to lake population, there was nevertheless overlap between lake populations in terms of multivariate space, particularly between Krasnyansky and Gluboke populations (Figure 2a). Examination of population structure revealed substantial admixture (Figure 2b), with 63% of individuals being assigned to the correct lake population. Finally, the likelihood of individuals being assigned to the correct population declined with increasing population radiation dose rate (GLM: χ^2^ = 11.94, *p* < 0.001; Figure 1e).

## 4 DISCUSSION

Ionizing radiation can cause mutations and thus supply genetic variation to populations (Adewoye et al., 2015; Parisot et al., 2017). This genetic variation is of fundamental importance, because it is the substrate for evolution and potentially adaptation within populations (Haldane, 1937; Kimura & Maruyama, 1966; Muller, 1927). On the contrary, radiation also has the potential to select against genotypes that are unable to cope with high radiation environments (Courtenay, 1965; Ellegren et al., 1997; Khodarev et al., 2004; Møller, 1993, 2002) and thus deplete variation. Previous work found that there was no evidence for phenotypic fitness differences among *Daphnia* populations that vary in contemporary radiation exposure across the CEZ (Goodman et al., 2019). However, this earlier finding does not mean radiation has no effect on populations. Other experimental studies have uncovered negative effects of ionizing radiation on fitness traits in both *Daphnia pulex* (Marshall, 1962) and *Daphnia magna* (Parisot et al., 2015). These works led us to hypothesize that evolutionary processes such as strong radiation‐mediated selection at the time of the accident (when dose rates were very high across the area) favoured the evolution of radiation‐tolerant *Daphnia* across populations, thus eliminating phenotypic differences between populations; the supply of *de novo* variation through mutation could subsequently be driving molecular evolution that is not (yet) visible at the level of the phenotype.

We found that lakes experiencing the highest radiation dose rates (Gluboke and Krasnyansky) contained *Daphnia* populations with the greatest population genetic diversity, consistent with radiation‐mediated supply of genetic variation (*sensu* Geras’kin & Volkova, 2014). This manifested as a positive relationship between dose rate and the average number of alleles per population (mean allelic richness; MAR; Figure 1a) and also expected heterozygosity (H_E_, Figure 1b). Moreover, our DAPC analysis uncovered that the lake populations that experience the highest radiation dose rates (Gluboke, Krasnyansky in particular) occupied the greatest volume of multivariate space, further supporting the idea of radiation‐mediated genetic variation (Figure 2a); these lakes also had the lowest likelihood of individuals being correctly assigned to their population based on genotype data alone (Figures 1e and 2b), with missassignment being most common between Krasnyansky and Gluboke populations. Other studies have revealed significantly higher mutation rates in microsatellite loci from samples within the CEZ experiencing contamination from the Chernobyl accident compared with local control sites (Baker et al., 2017; Dubrova et al., 1996; Ellegren et al., 1997; Kuchma et al., 2011). But ours is the first, to our knowledge, to show differences in population genetic diversity associated with continuous variation in dose rate across the Chernobyl area.

Microsatellites are neutral markers (Li et al., 2002), whereas selection acts directly on functional genes. As such, one must be careful not to over‐interpret patterns of selection using microsatellites. Nevertheless, since most mutations are deleterious, it is a reasonable assumption that radiation‐mediated selection, and thus genetic load, could correlate with the radiation dose experienced by populations. Any long‐term directional selection associated with chronic exposure would deplete genetic variation (Mort & Wolf, 1986), as genotypes with poor anti‐stress mechanisms are removed from high but not low dose populations (Diehn et al., 2009; Khodarev et al., 2004; Ramana et al., 1998). The positive relationship between radiation dose rate and genetic diversity (Figure 1a,b) demonstrates that any mutational supply likely outweighs genetic depletion due to radiation‐mediated selection in these Chernobyl *Daphnia* populations. This does not, of course, mean that radiation‐mediated selection is absent or weak. The positive relationships between population genetic diversity and radiation dose rate are driven by the two highest dose rate populations, and we cannot exclude a negative relationship between radiation dose rate and population genetic diversity at lower radiation levels (a limitation from having seven available populations). Further, it could be that radiation‐mediated selection for *Daphnia* with effective anti‐stress or DNA repair mechanisms was particularly strong at the time of the accident (Boubriak et al., 2008; Danchenko et al., 2009), acted on populations across the whole area, and that current radiation dose rates are below a threshold required to exert further selection. It is also possible that radiation‐mediated selection from lower dose chronic exposure is weaker and thus slower to act.

We next tested whether the Chernobyl accident may have indiscriminately culled genetic diversity within populations, driving genetic drift (Frankham et al., 2004), reducing the effective population size (Nei & Tajima, 1981). This is crucially important, as when effective population sizes are low, both beneficial and deleterious mutations behave as if they are neutral (Hartl & Clark, 1997), and there is little capacity for selection to drive adaptive evolutionary change (Hartl & Clark, 1997; Lande, 1993). As well as low diversity, genetic drift leads to increased differentiation among populations and strong population structure. We found little evidence for either. As discussed earlier, genetic diversity is surprisingly high (Figure 1a,b), and whereas populations are generally genetically distinct from each other (Table 2; Figure 2a), inter‐population differentiation is weak: only 12.5% of the overall genetic variation is due to between‐population differentiation (Table 2), overall *F*_ST_ is low, and there is overlap in the genetic characteristics of each lake (Figure 2). Together, these results provide evidence that genetic drift is not a strong force among Chernobyl *Daphnia* populations.

We further uncovered evidence of significant within‐population structure (*i*.*e*. inbreeding) in two populations (of intermediate radiation dose rate, Figure 1c), and no evidence for heterozygote excess. This is in stark contrast to the related *Daphnia magna*, where heterozygote excess is the norm and systematic inbreeding is either rare or completely absent (Haag et al., 2006; Hebert, 1974a, 1974b; Hebert & Ward, 1976; Walser & Haag, 2012). One possible reason for our findings is that the sex ratios varied among lake populations. The production of males in *Daphnia* populations is known to be determined by environmental change (such as increased population density, light levels or high levels of toxins) (Eads et al., 2008; Hobaek & Larsson, 1990), and biased sex ratios are known to cause inbreeding, though this is more of a risk in small populations (Mills & Smouse, 1994). Alternatively, and perhaps more likely, there could have been multiple episodes of hatching from the *Daphnia* resting egg bank within these three lakes and within seasons; this could have led to matings and thus sexual reproduction occurring within subsets of genotypes within lakes, thus facilitating within‐population structure (termed the Wahlund effect; see also Thielsch et al., 2009). In any case, radiation is unlikely to be driving either of these possible scenarios, as within‐population structure was not linked to dose rates.

It is important to consider the complex reproductive biology of *Daphnia* when evaluating population genetic structure. Genetic recombination is followed by a period of asexual reproduction, and asexual reproduction is often accompanied by clonal selection, where selection on any one trait involves selection on the whole genome (Lynch, 1987). Clonal selection means the same MLG is represented in multiple individuals (Halkett, 2005), *F*_IS_ values become negative within years as heterozygotes become overrepresented in the population and linkage disequilibrium can accumulate (this is in contrast to selection acting across bouts of sexual reproduction, which could generate positive *F*_IS_ values). We found only one instance, in Gluboke lake (which had the highest radiation dose rate), where the same MLG was collected twice and, as discussed earlier, there were no significantly negative *F*_IS_ values for that population. This could be because all the sampled lakes are sufficiently large to host very large *Daphnia* populations where the frequency of sex is high (Allen & Lynch, 2012). However, we did uncover signatures of linkage disequilibrium (${\bar{r}}_{D}$) and thus past clonal selection in Yampol, Buryakovka and Krasnyansky lakes. Importantly, the strength of this linkage disequilibrium was not associated with dose rate, suggesting that past bouts of clonal selection or sexual reproduction are caused by biotic or abiotic conditions that are unrelated to the nuclear accident.

Finally, we found evidence that gene flow from dispersal from neighbouring populations is reducing levels of population structure (in the form of strong isolation‐by‐distance: Figure 1d). High radiation environments could thus potentially supply genetic variation to other nearby populations as *Daphnia* resting stages disperse, fuelling within‐population evolution and adaptation in the manner of a metapopulations (Hanski, 1998). As such, it is plausible that the lack of phenotypic variation among contemporary Chernobyl *Daphnia* populations (Goodman et al., 2019) is likely concealing highly dynamic demographic and evolutionary processes that are, at least in part, fuelled by ionizing radiation.

It is important to note that this is a correlational study and not a common garden manipulation experiment, so we cannot completely exclude the idea that that an unmeasured variable could have shaped genetic diversity among these Chernobyl *Daphnia* populations. However, the relationships between radiation dose rate and measures of population genetic diversity are robust (Figure 1 and FigureS1), *in spite of* the low number of available populations, highly variable levels of linkeage disequilibrium and within‐population structure across lakes. This firmly points towards the explanation that *Daphnia* populations in high radiation lakes are experiencing greater mutation‐mediated supply of genetic variation than their low radiation lake counterparts. Of course, these findings provide the first insight into radiation‐mediated evolution in Chernobyl; the logical next step is to search for those mutations in coding regions and look for evidence of selection.

## CONFLICT OF INTEREST

The authors declare no conflict of interest.

## AUTHOR CONTRIBUTIONS

JG and SKJRA designed the research. JG conducted the *Daphnia* sampling. GL determined the radioisotope activities. JG calculated the radiation dose rates. JG and JB did the microsatellite genotyping. SKJRA contributed reagents and analytical tools. JG and SKJRA conducted the genetic analyses and wrote the manuscript. All authors approved the final version of the manuscript.

### PEER REVIEW

The peer review history for this article is available at https://publons.com/publon/10.1111/jeb.13983.

## Supporting information

Supplementary Material

## Data Availability

Microsatellite marker data are published on Dryad: https://doi.org/10.5061/dryad.0cfxpnw48.
