# Supplementary material for: Radiation‐mediated supply of genetic variation outweighs the effects of selection and drift in Chernobyl Daphnia populations
Source: J Evol Biol. 2022 Jan 29;35(3):413–22. doi: 10.1111/jeb.13983 (PMC9303301; doi:10.1111/jeb.13983)
Supplement: Supplementary file 1 — Supplementary Material [file JEB-35-413-s001.docx]

| **Table S1**: Information for the 10 microsatellite loci used for genotyping *Daphnia* samples. M = multiplex. | | | | | |
| --- | --- | --- | --- | --- | --- |
| Locus | Size range | NCBI accession number | Primer sequence (5’-3’) | M | Dye label |
| B008 | 150 - 175 | HQ234154 | F: TGGGATCACAACGTTACACAA  R: GCTGCTCGAGTCCTGAAATC | 1 | VIC |
| B030 | 150 - 176 | HQ234160 | F: CCAGCACACAAAGACGAA  R: ACCATTTCTCTCCCCCAACT | 1 | PET |
| B050 | 229 - 248 | HQ234170 | F: TTTCAAAAATCGCTCCCATC  R: TATGGCGTGGAATGTTTCAG | 1 | 6FAM |
| B064 | 135 - 155 | HQ234172 | F: CTCCTTAGCAACCGAATCCA  R: CAAACGCGTTCGATTAAAGA | 1 | 6FAM |
| B174 | 330 - 375 | HQ234205 | F: CATATTGGCACGACGTTCAC  R: GTTCCCTCATTCCCGATTTT | 2 | NED |
| B031 | 196 - 248 | HQ234161 | F: GTTGGCGCTGGCATATGTA  R: AAGAATTTTTGCAGCCGTTG | 2 | 6FAM |
| B075 | 124 - 150 | HQ234175 | F: GCTTGGGATCTCGAGAAGAA  R: ACTTGCTAGTGGCTGCTGCT | 2 | PET |
| B088 | 155 - 170 | HQ234179 | F: GGACAGTCGGCGTTCACT  R: CCTGTCGTGTTTTGATTTCCT | 2 | NED |
| B135 | 170 - 200 | HQ234191 | F: AAAGAGGGAGAATGTTGTTAGGC  R: TAAGGAGGGGGAAAAAGTGG | 2 | VIC |
| B155 | 290 - 321 | HQ234195 | F: GCGCATATGCAACAATTCAC  R: ACCTCCCCCTCACTTTGATT | 2 | PET |
|  |  |  |  |  |  |

|  |
| --- |
| Figure S1: Results from randomization tests demonstrating the robustness of the relationships between log10[radiation dose rate] and either A Mean allelic richness (MAR), or B Expected heterozygosity (H_exp_). |

| Table S2: Pairwise distances between lake populations (in Km). |
| --- |
| \|  \|  \|  \|  \|  \|  \|  \| \| --- \| --- \| --- \| --- \| --- \| --- \| --- \| \|  \| Smolin \| Yampol \| Glinka \| Buryakovka \| Krasnyansky \| Gluboke \| \| Vediltsy \| 22.31 \| 52.39 \| 67.15 \| 65.69 \| 52.83 \| 53.6 \| \| Smolin \|  \| 60.08 \| 76.57 \| 80.36 \| 68.99 \| 69.81 \| \| Yampol \|  \|  \| 16.71 \| 28.76 \| 26.87 \| 27.35 \| \| Glinka \|  \|  \|  \| 20.29 \| 26.88 \| 26.88 \| \| Buryakovka \| \|  \|  \|  \| 13.66 \| 13.06 \| \| Krasnyansky \| \|  \|  \|  \|  \| 0.82 \| \|  \|  \|  \|  \|  \|  \|  \| |
